# Paired-Like Homeobox 2B (PHOX2B) Mutation and the Hidden Endocrine Puzzle: Hyperinsulinism in Congenital Central Hypoventilation Syndrome

**DOI:** 10.7759/cureus.99114

**Published:** 2025-12-13

**Authors:** Mohamad Sabsabee, Manal Mustafa

**Affiliations:** 1 Pediatrics, Al Jalila Children's Speciality Hospital, Dubai, ARE; 2 Pediatric Endocrinology, Al Jalila Children's Speciality Hospital, Dubai, ARE

**Keywords:** cchs, congenital central hypoventilation syndrome, endogenous hyperinsulinism, hyperinsulinemic hypoglycemia, reactive hypoglycemia

## Abstract

Congenital central hypoventilation syndrome (CCHS) is a rare disorder of autonomic control of breathing caused predominantly by paired-like homeobox 2B (PHOX2B) mutations and frequently accompanied by broader autonomic dysfunction affecting cardiovascular, gastrointestinal, and endocrine systems. We report a four-month-old female with genetically confirmed PHOX2B polyalanine repeat expansion (c.726_764dup; p.Ala248_Ala260dup; 33 repeats) who developed recurrent, symptomatic postprandial hypoglycemia after transitioning from continuous enteral feeding to oral bolus feeds. Critical samples during hypoglycemia (blood sugar nadir 26 mg/dL) showed inappropriately elevated insulin and C-peptide with suppressed ketones and appropriate cortisol and growth hormone (GH) responses, while metabolic work-up was otherwise unremarkable. The pattern supported reactive postprandial hyperinsulinemic hypoglycemia due to autonomic dysregulation rather than congenital hyperinsulinism. Glycemic stability was achieved by reinstating slow, continuous feeds and avoiding rapid carbohydrate boluses; dextrose boluses precipitated rebound hypoglycemia. The case underscores an under-recognized endocrine manifestation of PHOX2B-related CCHS and highlights practical management-continuous/slow feeding, cautious use of dextrose, and multidisciplinary follow-up to maintain euglycemia during feeding transitions.

## Introduction

Congenital central hypoventilation syndrome (CCHS) is a rare genetic disorder--estimated incidence of one in 150,000 live births--characterized by failure of automatic control of breathing and widespread autonomic dysfunction [1,2]. It typically presents in the neonatal period with hypoventilation, particularly during sleep, but may also involve abnormalities of heart rate, gastrointestinal motility, temperature regulation, and pupillary responses [1,3]. The condition results almost exclusively from mutations in the paired-like homeobox 2B (PHOX2B) gene, which encodes a transcription factor essential for the development of autonomic and respiratory control centers in the brainstem [1,4].

PHOX2B mutations are broadly categorized as polyalanine repeat expansion mutations (PARMs) and non-polyalanine repeat mutations (NPARMs). The length of the polyalanine expansion correlates with disease severity: expansions of 20/25-27 repeats often present with milder, sleep-associated hypoventilation, whereas longer expansions (≥33 repeats) are associated with continuous ventilatory dependence and a higher risk of associated conditions, such as Hirschsprung disease, cardiac arrhythmias, and neural crest tumors [1,2,5]. The condition follows an autosomal dominant inheritance pattern with most cases arising de novo [6].

Although the primary clinical manifestation involves disordered ventilatory control, CCHS is increasingly recognized as a multisystem autonomic disorder. Endocrine manifestations, though uncommon, are emerging in the literature. Reported abnormalities include impaired glucose regulation, adrenal insufficiency, and disturbances in thermoregulation [3,7,8]. Hypoglycemia in this context is particularly significant because it may be misinterpreted as a metabolic disease or congenital hyperinsulinism. The underlying mechanism is believed to stem from autonomic dysfunction with impaired counter-regulatory hormone responses, altered insulin suppression, and abnormal parasympathetic tone [3,4,9]. This report describes a genetically confirmed infant with PHOX2B-related CCHS complicated by severe reactive hypoglycemia, highlighting the diagnostic challenges and management principles relevant to such presentations.

## Case presentation

A four-month-old female infant, born at term after an uneventful pregnancy, was diagnosed with congenital central hypoventilation syndrome (CCHS) based on clinical features and confirmatory genetic testing. Genetic analysis identified a heterozygous PHOX2B polyalanine repeat expansion mutation consistent with CCHS, as shown in Table 1.

**Table 1:** Genetic testing result. PHOX2B: paired-like homeobox 2B.

Gene	Variant	Zygosity	Classification	Disease	Inheritance
PHOX2B (MIM: 209880)	c.726_764dup (p.Ala248_Ala260dup); 33-polyalanine repeat expansion	Heterozygous	Pathogenic	Congenital central hypoventilation syndrome	Autosomal dominant

Neonatal course

Following delivery, the patient developed recurrent apnea, bradypnea, and desaturation episodes, necessitating non-invasive positive pressure ventilation (NIPPV) and eventual intubation. Cardiac evaluation revealed a patent foramen ovale (3-4 mm). TORCH screening was negative. She experienced neonatal seizures during the first month of life, managed with levetiracetam (Keppra). A trial of neostigmine was initiated for suspected congenital myasthenic syndrome but discontinued due to lack of improvement. Metabolic screening, including plasma amino acids, urine organic acids, and acylcarnitine profile, was unremarkable. She required a prolonged NICU stay due to recurrent apnea and ventilator dependence.

Respiratory and ventilatory management

Due to persistent central hypoventilation and recurrent apneic episodes, a tracheostomy was performed at eight weeks of age, and the patient was transitioned to home mechanical ventilation (volume control-synchronized intermittent mandatory ventilation (VC-SIMV) mode). Following stabilization, she was discharged to the pediatric ward for multidisciplinary discharge planning, including pulmonary, speech and language, neurology, ophthalmology, and dietetic assessments.

Feeding history and onset of hypoglycemia

Initially, the infant received continuous orogastric tube feeds of standard infant formula. After achieving clinical stability, the feeding regimen was gradually transitioned to bolus tube feeds every two hours and later to oral feeds after passing a swallowing study. However, during the transition from continuous to bolus oral feeding, she developed recurrent episodes of symptomatic hypoglycemia, characterized by lethargy and generalized tonic-clonic seizure.

She experienced a prolonged seizure associated with a blood glucose of 26 mg/dL (normal range: 60-180 mg/dL). Due to clinical suspicion of sepsis, a lumbar puncture performed at that time was positive for enterovirus, confirming viral meningitis. Subsequent glucose monitoring revealed recurrent, unpredictable hypoglycemia (nadir 26-36 mg/dL), often occurring one to two hours postprandially but not after fasting.

Laboratory evaluation

A critical sample collected during hypoglycemia demonstrated elevated insulin levels (up to 62 µIU/mL, normal range: 2.6-24.9 µIU/mL) and C-peptide concentrations (3.1 nmol/L, normal range: 0.37-1.47 nmol/L), with suppressed ketone levels (0.1 mmol/L, normal range: 0.0-0.6 mmol/L). Cortisol and growth hormone (GH) responses were adequate (cortisol: 486 nmol/L, normal range: 55-386 nmol/L; GH: 10 ng/mL, normal range: 0.12-7.79 ng/mL). Acylcarnitine and organic acid profiles were within normal limits, ruling out fatty acid oxidation defects. These findings supported a diagnosis of postprandial hyperinsulinemic hypoglycemia secondary to autonomic dysregulation associated with PHOX2B mutation rather than congenital hyperinsulinism.

Multidisciplinary management

Metabolic and endocrine consultations emphasized autonomic instability as the underlying mechanism. There was consensus on avoiding rapid dextrose boluses due to rebound hypoglycemia and recommending slow continuous feeds with close glucose monitoring. Diazoxide therapy was discussed but avoided due to the underlying mechanism of autonomic dysregulation and potential adverse effects, including fluid retention and pulmonary hypertension, and the absence of persistent fasting hyperinsulinemia.

Feeding was modified to continuous nasogastric infusion for two hours, followed by a one-hour rest. During rest periods, small volumes of thickened water were provided orally to maintain suckling ability. This regimen resulted in stabilization of glucose levels, with no further symptomatic hypoglycemic episodes.

Discharge and follow-up

The patient was discharged home on mechanical ventilation and nasogastric feeding, with comprehensive caregiver training and scheduled follow-ups by the multidisciplinary team. She remained clinically stable with no recurrent hypoglycemia on a continuous feeding regimen. Psychological support was provided to the family, and discussions were initiated regarding the eventual placement of a gastrostomy for long-term nutritional management.

## Discussion

Mechanism and literature review

PHOX2B is a transcription factor essential for the development of autonomic circuits integrating respiratory and metabolic control [1,4]. Disruption leads to attenuated sympathetic outflow and impaired counter-regulatory hormone responses (glucagon, cortisol, GH), alongside failure to suppress insulin appropriately after carbohydrate loads [4,9]. Case series and reports have described CCHS associated with hypoglycemia--often hyperinsulinemic and postprandial--supporting an autonomic rather than primary pancreatic mechanism. Farina et al. (2012) reported three cases of CCHS with hypoglycemia, in which seizures were the presenting symptom; postprandial hypoglycemia was observed in the three patients despite prolonged fasting tolerance. Glucagon and diazoxide treatments were initiated along with dietary intervention [3]. More recently, Malhotra et al. (2024) reported six cases of CCHS, four of which presented with postprandial hypoglycemia, while the others had fasting hypoglycemia. Elevated insulin with suppressed ketones was observed during these episodes, and treatment with diazoxide and dietary modification was successful. The dietary intervention focused mainly on avoiding rapid bolus feeds and shifting to a slower continuous feeding regimen [8]. It is worth noting that most of these patients showed improvement over time and were weaned off diazoxide therapy by three to five years of age. Similarly, in our patient, hyperinsulinemia with suppressed ketogenesis and appropriate counter-regulatory hormone response during hypoglycemia, normal acylcarnitines/organic acids, and a non-elevated glucose requirement index together argued against congenital hyperinsulinism and for autonomic-mediated reactive hypoglycemia [10].

Implications for management

Current CCHS guidance emphasizes comprehensive autonomic care and nutrition strategies [2,5]. For hypoglycemia suspected to be reactive/autonomic in origin, practical steps include: (i) avoid rapid carbohydrate boluses and IV dextrose pushes when clinically safe; these can trigger rebound hypoglycemia; if an acute dextrose bolus is required, follow with continuous infusion or slow feeds [10]; (ii) prefer continuous or slow enteral delivery with careful titration during transitions [5]; (iii) consider diazoxide only if conservative measures fail, as glucose requirement was not elevated (in contrast to congenital hyperinsulinism) [10]; and (iv) implement frequent or continuous glucose monitoring with point-of-care-testing (POCT) confirmation of low values [11].

Rationale for avoiding diazoxide. Diazoxide acts on adenosine triphosphate (ATP)-sensitive potassium channels in pancreatic β-cells to suppress insulin release [12]. While effective in congenital hyperinsulinism, its use in autonomic-mediated hypoglycemia is limited because insulin secretion is not persistently excessive [10]. Unnecessary diazoxide may cause fluid retention, pulmonary hypertension, hypertrichosis, gastrointestinal irritation, and potential cardiac failure in infants [12,13]. In CCHS, pulmonary hypertension risk and ventilatory dependence make these adverse effects particularly hazardous [13,14]. Recent reviews highlight diazoxide-associated pulmonary hypertension occurring in up to 7-8% of treated infants, often within the first two weeks of therapy and reversible upon discontinuation [13,14]. Therefore, diazoxide is reserved for refractory cases in which persistent biochemical hyperinsulinism has been proven, and conservative feeding strategies have failed.

What this case adds

This infant with a 33-alanine PHOX2B expansion had reproducible postprandial hyperinsulinemic hypoglycemia precipitated by bolus feeds and mitigated by slow continuous feeds, aligning with autonomic dysregulation models [3,8]. The course can be further complicated by intercurrent illnesses amplifying autonomic imbalance, highlighting the need for heightened surveillance during infections in CCHS [7].

## Conclusions

CCHS, due to a PHOX2B mutation, can manifest with reactive hypoglycemia from autonomic-endocrine dysregulation. Elevated insulin and C-peptide with low ketones during hypoglycemia indicate functional, not structural, hyperinsulinism. Slow continuous feeding and avoidance of carbohydrate boluses are effective management strategies. Diazoxide should be avoided unless persistent hyperinsulinism is proven, due to the risk of pulmonary hypertension and fluid retention. Awareness of this presentation can prevent misdiagnosis and harmful interventions.
